# Survey of Time Series Data Generation in IoT

**DOI:** 10.3390/s23156976

**Published:** 2023-08-05

**Authors:** Chaochen Hu, Zihan Sun, Chao Li, Yong Zhang, Chunxiao Xing

**Affiliations:** 1 Beijing National Research Center for Information Science and Technology, Tsinghua University, Beijing 100084, China; hcc20@mails.tsinghua.edu.cn (C.H.); sunzh22@mails.tsinghua.edu.cn (Z.S.); xingcx@tsinghua.edu.cn (C.X.); 2 Department of Computer Science and Technology, Tsinghua University, Beijing 100084, China

**Keywords:** time series, data generation, categorization, IoT

## Abstract

Nowadays, with the rapid growth of the internet of things (IoT), massive amounts of time series data are being generated. Time series data play an important role in scientific and technological research for conducting experiments and studies to obtain solid and convincing results. However, due to privacy restrictions, limited access to time series data is always an obstacle. Moreover, the limited available open source data are often not suitable because of a small quantity and insufficient dimensionality and complexity. Therefore, time series data generation has become an imperative and promising solution. In this paper, we provide an overview of classical and state-of-the-art time series data generation methods in IoT. We classify the time series data generation methods into four major categories: rule-based methods, simulation-model-based methods, traditional machine-learning-based methods, and deep-learning-based methods. For each category, we first illustrate its characteristics and then describe the principles and mechanisms of the methods. Finally, we summarize the challenges and future directions of time series data generation in IoT. The systematic classification and evaluation will be a valuable reference for researchers in the time series data generation field.

## 1. Introduction

A time series is a form of data that records events or quantities occurring over time, usually indexed by timestamps. They can be sampled periodically or irregularly and cover various fields such as sensor readings, financial market quotes, weather forecasts, etc. Mathematically, a time series comprises a series of ordered data points, where each data point represents a value or state at a certain point in time. A time series can be expressed as a sequence, denoted as $\left(t_{1},x_{1}\right),\left(t_{2},x_{2}\right),\ldots ,\left(t_{n},x_{n}\right)$, where $t_{i}$ represents the timestamp, which can be discrete or continuous, of the *i*th data point and $x_{i}$ represents the value of the *i*th data point. A time series usually contains certain time correlations and regular characteristics of the sequence itself, such as periodicity, trend, seasonality, etc. These characteristics can be mined and analyzed through time series analysis, signal processing, machine learning and other methods.

**Figure 1:**
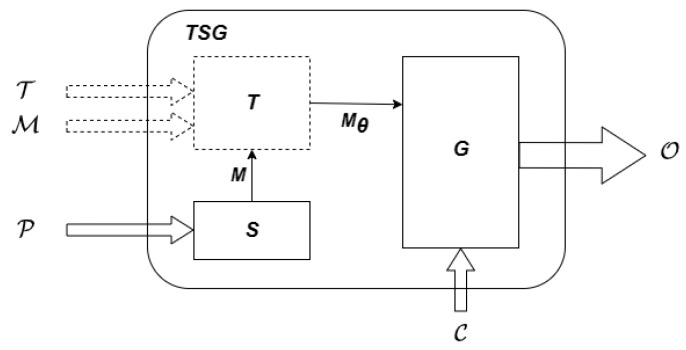
A formal model of time series data generation methods.

Time series data generation refers to the use of specific methods and technologies to generate time series data that conform to specific rules or patterns, which can effectively solve these problems. As shown in Figure 1, a time series data generation method TSG=(S,T,G) contains a model selector *S*, a model trainer *T*, and a generator *G*. The selector *S* takes prior knowledge P as input and selects a generative model *M*. The trainer *T* takes real time series data T, metadata M, and the selected model *M* as input, and obtains a trained model with parameters $M_{\theta }=T\left(T,M,M\right)$. The generator *G*, finally, generates new time series data O using the trained model $M_{\theta }$ and control information C. The graphics made up of dashed lines represent optional components.

With the development of IoT technology [1] more and more sensors are being deployed in various fields, ranging from industrial manufacturing and transportation to medical care. This development results in the constant generation of a large amount of time series data. These time series data can come from various fields, including architecture [2,3], meteorology [4], finance [5], transportation [6], medical treatment [7], biomedical signals [8], environmental pollution [9], earthquake geology [10], etc. They reflect various phenomena and events that change over time. At the same time, the complexity of time series data is also increasing. The massive volume and complexity bring greater challenges to data analysis. To address this, various technologies are employed to manage [11], store [12], process, and analyze [13] time series data aiming to extract useful information from the data.

Time series data plays an important role in scientific and technological research. Through the analysis of time series data, it is possible to reveal the underlying patterns and laws in the data, discover the correlation and periodicity between events, and then deeply understand the nature and mechanism of the event itself, providing strong support for research in related disciplines. Specifically, a deep understanding of time trends [14], periodicity [15,16], correlation [17,18], etc., can be gained and valuable information can be further extracted, such as anomaly detection [19,20,21], classification [22,23,24], clustering [25,26], etc. These studies require a large amount of time series data for experiments to test the effectiveness and practicality of different algorithms and techniques, optimize the parameters and structure of algorithms, evaluate the performance and accuracy of different techniques, and train machine learning models.

However, there are two challenges in acquiring massive time series data. First, publicly obtainable time series data is limited because it may contain sensitive or confident information. For example, data from sensors may leak information such as location and temperature [27,28]. Second, due to the diversity of data sources, the instability of data collection, noise, etc., the quality of time series data is often relatively low, so it needs to be cleaned and verified. Low quality may bring various problems. For the problem of an unbalanced data distribution, some data sets may have unbalanced distribution problems, which will lead to a decrease in the performance of the training model. For the data diversity problem, datasets in some domains may lack diversity to cover all scenarios and situations.

Time series data generation can solve the two challenges above. First, by generating synthetic data, privacy is preserved and data sharing and analysis is allowed. At the same time, it is possible to expand the size of the dataset. Second, the data quality issues can be reduced or eliminated, resulting in more accurate and high-quality data. At the same time, the coverage of the data sets can be expanded, making the data more balanced and diverse, and improving the performance of the model. As a result, time series analysts, time series researchers, and time series processing system and database testing engineers will benefit from time series generation.

Researchers in many fields have proposed a variety of time series data generation methods in their respective fields such as biology [8], database benchmark [29,30], electricity [31], energy [32,33,34,35,36,37,38,39], environment [40,41,42], finance [5,43], medicine [7], music [44], networks [45], remote sensing [46,47,48,49] and sensors [50]. Despite the abundance of research on time series generation, a comprehensive survey that systematically classifies and evaluates the previous work is lacking. Researchers may find it hard to select appropriate generation methods for different scenarios. This survey aims to bridge this gap.

The sources of articles that are taken into consideration are top journals such as those published by IEEE, Springer, Elsevier, etc., and proceedings of top conferences such as AAAI, ICDM, NeurIPS, VLDB, etc. The keywords used for the search strategy are “time series generation”, “temporal data generation”, “time series prediction”, “sequence generation”, “series GAN”, and “series VAE”. We selected the articles related to time series generation and took their common references into consideration. The commonly cited articles and the new articles with sufficient novelty are selected for our survey.

Based on the underlying algorithms and models used by the existing time series data generation methods, this paper divides these methods into four categories: rule-based methods [51,52,53], simulation-model-based methods [37,45], traditional machine-learning-based methods [29,32,39] and deep-learning-based methods [7,30,44,54,55,56,57,58,59,60,61,62,63]. Rule-based methods use a set of rules or constraints to specify the properties and structure of the data to be generated. A simulation-model-based method refers to the establishment of simulation models to simulate various situations in real systems or events, thereby generating time series data. Traditional machine-learning-based methods typically utilize classic machine learning algorithms to generate time series data. These algorithms leverage existing time series data as the training datasets, learn the characteristics and patterns of the time series data, train the generative models, and use these models to generate new time series data. Deep-learning-based methods rely on deep neural network models to generate time series data. They typically use models such as generative adversarial networks (GANs) [64] or variational autoencoders (VAEs) [65] to generate data with specific temporal dependencies.

The key contributions of this paper are summarized as follows:

1. The time series data generation methods are classified into four categories based on their underlying algorithms and models.
2. The characteristics, mechanisms and application scenarios of each category are illustrated.
3. The challenges and future directions of time series data generation are summarized.

This paper first introduces the four categories of time series data generation methods with an analysis of their pros and cons in Section 2, then introduces the methods of each category in detail from Section 3, Section 4, Section 5 and Section 6. After that, this paper summarizes the challenges and future directions of time series data generation in Section 7. Finally, the conclusions are presented in Section 8.

## 2. Four Categories of Time Series Data Generation Methods

Based on the underlying algorithms and models used by time series data generation methods, they can be divided into the following four categories. These methods have several features, of which the need for real data and domain-specific knowledge describe the difficulty of training/constructing the underlying models; the authenticity and complexity of generation results describe the quality of generated time series data; the controllability of results is an important feature describing how much the users can take control of the methods to obtain the desired results. These features of the four categories of methods are summarized in Table 1.

Rule-based methods: These use a set of rules or constraints to specify the properties and structures of the data to be generated. These rules can be based on properties of the data such as data type, data range, data density, etc. In addition, rules can also be based on relationships between data, such as correlations and dependencies, and so on. These rules are used to specify the required data attributes, ensuring that the generated data will conform to these rules and restrictions. These methods do not need to rely on large amounts of historical data or training models; the generated data may be relatively simple and unrealistic.

Simulation-model-based methods: These approaches use computer simulation techniques to generate time series data based on the modeling of actual scenarios or systems. For example, a fluid dynamics model or a mechanical model may be used to generate corresponding time series data. These methods can generate more realistic data, simulate the behavior of complex systems, and produce different data by changing model parameters, but they require a large amount of domain knowledge and model parameters. Additionally, the amount of calculations is relatively large.

Traditional machine-learning-based methods: These methods are based on traditional machine learning algorithms, which utilize existing time series data to train the models, and then generate new time series data using the models. For example, time series data can be generated using algorithms such as linear regression, support vector machines, or random forests. These methods take into account the influence of historical data, but require parameter adjustment and model training.

Deep-learning-based methods: These methods are based on deep learning algorithms and use deep learning models such as recurrent neural networks (RNNs) or convolutional neural networks (CNNs) to generate time series data. For example, models such as GANs or VAEs can be used to generate time series data. These methods can generate more complex data and take into account the impact of longer time spans, but require a large amount of training data and computing resources.

As can be seen from the features shown in Table 1, rule-based methods can be used for scenarios in which the distribution of the generated data does not depend on the real data; simulation-model-based methods can be used for scenarios in which the distribution of the generated data is the same as a known stochastic process in the real world; traditional machine-learning-based and deep-learning-based methods can be used for scenarios in which the distribution of the generated data is unknown beforehand and can be learned from the real data.

## 3. Rule-Based Methods

This section describes rule-based methods. Section 3.1 introduces three common time series models. Section 3.2 introduces three rule-based methods to generate data. The first two methods are proposed for the data generation of traditional relational databases, while the third solution uses the MAR model, which provides users with a wealth of adjustable parameters.

The characteristics of rule-based methods are as follows:

1. Simplicity and speed: Rule-based methods are simple, only need to define rules and parameters, and use random number generation. Therefore, the generation speed is fast, which can meet some scenarios with high real-time requirements.
2. Strong controllability: The properties of generating time series data can be easily controlled and adjusted using rule-based methods. By modifying the rules and parameters, data that meet specific needs can be easily generated.
3. Low reliance on historical data: Rule-based methods do not require a large amount of historical data to generate new data. This makes them useful in situations where historical data are scarce.
4. Lack of authenticity: Since the generated data are based on fixed rules and parameters, without considering the actual system behavior, they may be different from the actual data and lack authenticity.
5. Inability to simulate complex system behavior: These methods are generally unable to simulate complex system behavior, because the actual system behavior is often very complex and cannot be well described and simulated by simple rules and parameters.

### 3.1. Common Time Series Models

#### 3.1.1. Autoregressive (AR) Model [66]

An AR model of order *p*, denoted as AR(p), can be defined as:

(1) $$x_{t}=\sum_{i=1}^{p}\phi_{i}x_{t-i}+\epsilon_{t}$$

where $\phi_{1},\ldots ,\phi_{p}$ are the parameters of the model, and $\epsilon_{t}$ is a white noise, whose samples are regarded as a sequence of serially uncorrelated random variables with zero mean and finite variance, thus providing the randomness.

#### 3.1.2. Moving-Average (MA) Model [67]

An MA model of order *q*, denoted as MA(q), can be defined as:

(2) $$x_{t}=\mu +\sum_{i=1}^{q}\theta_{i}\epsilon_{t-i}+\epsilon_{t}$$

where μ is the mean of the series, the $\theta_{1},\ldots ,\theta_{q}$ are the parameters, and $\epsilon_{t},\epsilon_{t-1},\ldots ,\epsilon_{t-q}$ are white noise error terms.

#### 3.1.3. Autoregressive Moving-Average (ARMA) Model [68]

An ARMA model with *p* autoregressive terms and *q* moving-average terms is denoted as ARMA(p,q). This model contains the AR(p) and MA(q) models:

(3) $$x_{t}=\epsilon_{t}+\sum_{i=1}^{p}\phi_{i}x_{t-i}+\sum_{i=1}^{q}\theta_{i}\epsilon_{t-i}$$

#### 3.1.4. Autoregressive Integrated Moving-Average (ARIMA) Model [69]

An ARIMA model is a generalization of an ARMA model. An ARIMA model of AR order *p*, MA order *q*, and a degree of differencing *d*, denoted as ARIMA(p,d,q), can be defined as:

(4) $$\left(1-\sum_{i=1}^{p}\phi_{i}L^{i}\right){\left(1-L\right)}^{d}x_{t}=\left(1+\sum_{j=1}^{1}\theta_{j}L^{j}\right)\epsilon_{t}$$

where *L* is the lag operator, $L^{i}x_{t}=x_{t-i}$.

### 3.2. Rule-Based Methods

#### 3.2.1. FDG [51]

Obtaining comprehensive real data can be difficult, and without a flexible data generation framework capable of modeling various rich data distributions, real data may not be available at all, or it may not be comprehensive enough to thoroughly evaluate the system under consideration. This work proposes a flexible database generation framework, introduces a data generation language (DGL), uses iterators as basic units to form data tuple generation streams, and applies it to generate databases with complex composite distributions and inter-table dependencies.

#### 3.2.2. SRDG [52]

SRDG is a general-purpose relational data generation tool designed for database testing. It supports the definition of relationships within and between tables, and users can specify some simple data characteristics. The data generation algorithm is based on a graph model, in which tables are represented as nodes and foreign-key constraints as edges. The generation algorithm is a depth-first traversal which begins at non-referenced nodes and then examines all out-bound edges, generating data according to the types of the edges.

#### 3.2.3. GRATIS [53]

Generating time series (GRATIS) is a time series data generator that utilizes the mixture autoregressive (MAR) model [70]. The generator is designed for testing various time series analysis methods and provides diverse parameters to efficiently generate new time series data with controllable features. A *K*-component MAR model, which is actually a finite mixture of *K* Gaussian AR models, can be defined as:

(5) $$F\left(x_{t}|F_{-t}\right)=\sum_{k=1}^{K}\alpha_{k}\Phi \left(\frac{x_{t}-\phi_{k0}-\phi_{k1}x_{t-1}-\ldots -\phi_{kp_{k}}x_{t-p_{k}}}{\sigma_{k}}\right)$$

where $F(x_{t}|F_{-t})$ is the conditional cumulative distribution of $x_{t}$ given the past information $F_{-t}\subseteq \left\{x_{t-1},\ldots ,x_{t-p_{k}}\right\}$, Φ(·) is the cumulative distribution function of the standard normal distribution, $x_{t}-\phi_{k0}-\phi_{k1}x_{t-1}-\ldots -\phi_{kp_{k}}x_{t-p_{k}}$ is the autoregressive term in each mixing component, $\sigma_{k}>0$ is the standard error, $\sum_{k=1}^{K}\alpha_{k}=1$, and $\alpha_{k}>0$ for k=1,2,…,K.

To generate diverse time series data instances, it uses distributions instead of fixed values for the parameters in the underlying models. It also adopts a genetic algorithm to tune the MAR model parameters to generate time series data with target features extracted from real time series data.

## 4. Simulation-Model-Based Methods

This section introduces two methods for time series data generation by constructing simulation models. These two methods select specific simulation models for the workload of the cloud data center and wind speed.

The characteristics of simulation-model-based methods are as follows:

1. Ability to simulate actual system behavior: Compared with the rule-based methods, simulation-model-based methods can simulate the behavior of actual systems more accurately, because the simulation models can analyze and model the actual system behavior and simulate the dynamic evolution of the system.
2. Interpretability of generated data: Through the analysis and adjustment of the simulation model, the reasons and rules of the generated data can be well explained, making the generated data more reliable and interpretable.
3. Reliance on model accuracy: The accuracy of the generated data is closely related to the accuracy of the model. If the accuracy of the model is not high, errors and deviations may also exist in the generated data.
4. Slow data generation speed: Compared with the rule-based random generation methods, simulation-model-based methods require model building, so the generation speed is relatively slow and cannot meet the high real-time requirements of the scene.
5. Limited data quantity: Due to the need of model building, a certain amount of historical data are required for training and adjustment of the model. Therefore, when the amount of data is insufficient, the accuracy and reliability of the generated data will be affected.

Kultok et al. [45] proposed a model-based method to create synthetic workload trajectories for cloud data centers. It randomly samples from existing time series data, selects some alternative distributions, and calculates the parameters of these distributions using the maximum likelihood method. After that, the Anderson–Darling test is used to select a suitable distribution from the alternative distributions, and the initial data set is randomly sampled from the selected distribution. Then, it runs an iterative process to rearrange the initial data set. In each iteration, the current series is shuffled, and if it has a smaller mean square error (MSE) than the real time series data, the outcome is selected as the current series. The process continues until the current series reaches an MSE that is less than a threshold.

Bokde et al. [37] proposed two generation methods for synthesizing wind speed time series data. Both of the methods first sample the initial data from a Weibull distribution. The Weibull distribution [71] is parameterized by x,k and its probability density function is

(6) $$f\left(x;\lambda ,k\right)=\left\{\begin{array}{ccc} \frac{k}{\lambda }{\left(\frac{x}{\lambda }\right)}^{k-1}e^{-{\left(x/\lambda \right)}^{k}} & \; & x\geq 0\\ 0 & \; & x<0 \end{array}\right.$$

The methods then rearrange them to make their autocorrelation properties close to the real data, which is the difference between these two methods. The first method arranges the data so that the order of the generated series data is the same as the order of the existing data. In contrast, the second method calculates the parameters of the Weibull distribution based on the existing data, samples the distribution to obtain a batch of data, and selects values whose distances to the real data are less than a threshold in order. The process is repeated until a time series of a given length is generated.

## 5. Traditional Machine-Learning-Based Methods

This section introduces traditional machine-learning-based methods. Section 5.1 introduces the traditional Markov model. Then, Section 5.2 presents three time series data generation methods based on variants of the Markov model.

The characteristics of the methods of generating time series data based on traditional machine learning are as follows:

1. Ability to process data with more complex patterns: Traditional machine-learning-based methods can process data with more complex patterns, enabling them to better capture the complex relationships between data and generate more realistic data.
2. Fast data generation: The methods have fast data generation speeds, so they can produce large amounts of data in a short period of time.
3. Rely on model accuracy: The accuracy of the generated data is closely related to the accuracy of the model. If the accuracy of the model is not high, errors and deviations may exist in the generated data.
4. Sufficient historical data required: Due to the need to train the model, a certain amount of historical data are necessary for the training and adjustment of the model. Therefore, when the amount of data is insufficient, the accuracy and reliability of the generated data will be affected.

### 5.1. Markov Model

#### 5.1.1. Discrete-Time Markov Chain [72]

A discrete-time Markov chain is a stochastic process that can be parameterized by empirically estimating transition probabilities between discrete and finite states. The state at time step *i* is a random variable $X_{i}$ and the probability of moving to the next state depends only on the present state but not on the previous states:

(7) $$Pr\left(X_{i+1}=x_{i+1}|X_{1}=x_{1},\ldots ,X_{i}=x_{i}\right)=Pr\left(X_{i+1}=x_{i+1}|X_{i}=x_{i}\right)$$

This probability is called the transition probability from state $x_{i}$ to $x_{i+1}$. All of the possibilities between the states form a transition matrix.

#### 5.1.2. Hidden Markov Model (HMM) [73]

A hidden Markov model is a statistical Markov model that consists of a hidden state *X* and an observable state *Y*. Formally, let $X_{i}$ and $Y_{i}$ be discrete-time stochastic processes and i≥1. $X_{n}$ is a Markov process whose behavior is not directly observable. The state of *Y* at step *i* is determined only by the state of *X* at step *i*:

(8) $$P\left(Y_{i}=y_{i}|X_{1}=x_{1},\ldots ,X_{i}=x_{i}\right)=P\left(Y_{i}=y_{i}|X_{i}=x_{i}\right)$$

This probability is called the emission probability.

### 5.2. Traditional Machine-Learning-Based Methods

IoTAbench [29] is a benchmark toolkit designed for IoT big data scenarios. It contains a Markov chain-based synthetic data generator for smart meter data. The generator can learn the statistical properties from real time series data. To capture the dependence on several contextual features such as time of day, weather, etc., and incorporate them into the model, the generator augments the Markov chain model by adding additional inputs. It uses maximum likelihood estimation to estimate the transitional probability matrix from the empirical data. It also employs Laplace smoothing, which increases the count for each transition by one, to address the sparse problem of the transitional probability matrix.

Shamshad et al. [32] proposed a method which uses a probability transition matrix of first-order and second-order Markov chains to synthesize new data from existing wind speed data. Each state in the Markov chain represents a wind speed range.

Li et al. [39] proposed a method which uses the Gaussian mixture model hidden Markov model to generate medium- and long-term wind power generation data. The method uses the expectation-maximum (EM) [74] algorithm to estimate the parameters of the model, and then randomly samples from the initial state probability distribution to generate an initial hidden state. It generates a random number according to the uniform distribution on the (0,1) interval, and finds a hidden state that conforms to the state probability transition matrix according to the random numbers as the hidden state at next time step. To generate time series data from the hidden states, it converts the hidden states into the arguments of the Gaussian mixture model and samples the time series data from the model.

## 6. Deep-Learning-Based Methods

This section describes deep-learning-based methods. Section 6.1 introduces GANs, and lists several methods that use GANs to generate time series data. Most of these methods use the combination of a GAN and an RNN. Some of the methods also support conditional input. The features of GAN-based methods are summarized in Table 2. Afterwards, Section 6.2 introduces VAEs and time series data generation methods based on them.

The methods of generating time series data based on deep learning have the following features:

1. Learning higher-level features: The deep generative models can learn higher-level features and can automatically capture nonlinear and complex relationships in the data, thereby generating more realistic and complex data.
2. Generating more diverse data: The methods can not only generate data with similar characteristics, but also generate more diverse data, which allows patterns of data to be shown from various angles. This enables a better data generalization ability.
3. Generating more realistic data: The deep generative models can learn the high-order statistical features of data, thereby generating more realistic data, and the differences between the data generated by the model and the real data are becoming smaller and smaller.
4. Difficulty in training: Compared with traditional machine learning models, the training processes of deep generative models are more complicated and require more computing resources and time.
5. High data volume: Deep generation models require large amounts of data for training. If the amount of training data is insufficient, the generalization ability of the model and the accuracy of the generated data will be affected.

### 6.1. GAN-Based Methods

Generative Adversarial Network [64]:

A generative adversarial network (GAN) is a deep learning model designed by Ian J. Goodfellow et al. Given a training set, this technique learns to generate new data with the same statistics as the training set. It consists of two adversarial parts: a generative model *G* that captures the data distribution and a discriminative model *D* that estimates the probability that a sample came from the training data rather than *G*. *D* is trained to maximize the probability of assigning the correct label, while *G* is trained to minimize the probability of being discriminated. The adversarial parts play a two-player minimax game with value function V(G,D):

(9) $$\underset{G}{min}\underset{D}{max}V\left(D,G\right)=E_{x∼p_{data}\left(x\right)}\left[logD\left(x\right)\right]+E_{z∼p_{z}\left(z\right)}\left[log\left(1-D\left(G\left(z\right)\right)\right)\right]$$

where *x* is the real data subject to distribution $p_{data}\left(x\right)$ and *z* is an input noise variable of *G* subject to distribution $p_{z}\left(z\right)$.

#### 6.1.1. C-RNN-GAN [44]

Continuous RNN-GAN (C-RNN-GAN) is a recurrent neural network architecture that is trained with adversarial training to model the whole joint probability of a sequence, and to be able to generate sequences of data. The recurrent network used in the discriminator is long short-term memory (LSTM) [75]. The model is evaluated by learning the generating distribution behind classical music so the signal at every data point is modeled with four real-valued scalars: tone length, frequency, intensity, and time spent since the previous tone.

#### 6.1.2. RCGAN [7]

The recurrent conditional generative network (RCGAN) utilizes a GAN where the generator and discriminator are substituted by recurrent neural networks. The generator of RCGAN accepts a random seed and auxiliary condition input at each step, and the discriminator accepts the output of the generator and the auxiliary condition as input. LSTM was chosen as the implementation of RNN. The maximum mean discrepancy (MMD) was used to evaluate the authenticity of the data generated by the algorithm. This work also proposes a “train on synthetic data, test on real data” (TSTR) approach to evaluate generative algorithms. The process involves training a classifier using the data generated by the algorithm and testing the performance of the classifier on real data to represent the performance of the generated algorithm.

#### 6.1.3. SeqGAN [54]

SeqGAN models the data generator as a stochastic policy in reinforcement learning (RL) [76] and bypasses the generator differentiation problem by directly performing gradient policy updates. The RL reward signal that comes from the GAN discriminator is evaluated on a complete sequence and passed back to the intermediate-state action steps using a Monte Carlo search [77]. SeqGAN first pre-trains the generator $G_{\theta }$, parameterized by θ using maximum likelihood estimation, and uses its output to pre-train the discriminator $D_{\phi }$, parameterized by ϕ via minimizing the cross-entropy. It then starts adversarial training iteratively. In each iteration, $G_{\theta }$ first generates a sequence $Y_{1:T}=\left(y_{1},\ldots ,y_{T}\right)$ and computes an action value $Q(a=y_{t},s=Y_{1:t-1})$ for each $y_{t}$ using the following equation:

(10) $$Q_{D_{\phi }}^{G_{\theta }}\left(a=y_{t},s=Y_{1:t-1}\right)=\left\{\begin{array}{ccc} \frac{1}{N}\sum_{n=1}^{N}D_{\phi }\left(Y_{1:T}^{n}\right),Y_{1:T}^{n}\in MC^{G_{\beta }}\left(Y_{1:t};N\right) & \; & \mathrm{for}\;t<T\\ D_{\phi }\left(Y_{1:t}\right) & \; & \mathrm{for}\;t=T \end{array}\right.$$

where $MC^{G_{\beta }}\left(Y_{1:t};N\right)$ is a Monte Carlo search with a roll-out policy $G_{\beta }$ to sample the unknown last T−t tokens. After computing the action value, it updates generator parameters with the following gradient:

(11) $$\nabla_{\theta }J\left(\theta \right)≃\sum_{t=1}^{T}E_{y_{t}∼G_{\theta }\left(y_{t}|Y_{1:t-1}\right)}[\nabla_{\theta }logG_{\theta }\left(y_{t}|Y_{1:t-1}\right)\cdot Q\left(y_{t},Y_{1:t-1}\right)]$$

Then, it uses the current $G_{\theta }$ to generate negative examples, combines them with given positive examples *S*, and trains $D_{\phi }$. SeqGAN repeats the iterations until it converges. It adopts LSTM and CNN as the implementation for the generator and discriminator, respectively.

#### 6.1.4. T-CGAN [55]

The time conditional generative adversarial network (T-CGAN) is based on conditional generative adversarial networks (CGANs) [78], where the generator is implemented by a deconvolutional neural network and the discriminator is implemented by a convolutional neural network (CNN) [79]. Both the generator and the discriminator are conditioned on the sampling timestamps. This method is primarily used to augment data for time series with irregular sampling. The objective function of this model is:

(12) $$\underset{G}{min}\underset{D}{max}V\left(D,G\right)=E_{x∼p_{data}\left(x\right)}\left[logD\left(x|t\right)\right]+E_{z∼p_{z}\left(z\right)}\left[log\left(1-D\left(G\left(z|t\right)\right)\right)\right]$$

where $t=<t_{1},\cdots ,t_{n}>$ is a sorted vector of timestamps sampled at random from a space *T*.

The generator consists of four deconvolution layers with ReLU activation functions and batch normalization at each layer except for the last one. The discriminator is composed of two layers of convolution, each followed by a max-pooling layer and at the end there is a fully connected layer.

#### 6.1.5. TimeGAN [56]

The time series generative adversarial network (TimeGAN) is the first to combine the flexibility of the unsupervised GAN framework with the control afforded by supervised training in autoregressive models. It adopts the original unsupervised adversarial loss as well as a stepwise supervised loss using the real data as supervision, thereby explicitly encouraging the model to capture the stepwise conditional distributions in the data. Moreover, it utilizes an embedding network to provide a reversible mapping between features and latent representations, thereby reducing the high-dimensionality of the adversarial learning space. It divides the features of time series data into static features S∈S and temporal features X∈X. TimeGAN consists of four network components: embedding functions $e_{S}:S\to H_{S}$, $e_{X}:H_{S}\times H_{X}\times X\to H_{X}$, recovery functions $r_{S}:H_{S}\to S$,$r_{X}:H_{X}\to H_{S}\times H_{X}\times X$, sequence generators $g_{S}:Z_{S}\to H_{S}$, $g_{X}:H_{S}\times H_{X}\times Z_{X}\to H_{X}$, and sequence discriminators. The embedding network provides the latent space, the adversarial network operates within this space, and the latent dynamics of both real and synthetic data are synchronized through a supervised loss. Therefore, the object function contains three parts.

Reconstruction loss:

(13) $$L_{R}=E_{s,x_{1:T}∼p}[\|s-\tilde{s}{\|}_{2}+\sum_{t}{\left\|x_{t}-{\tilde{x}}_{t}\right\|}_{2}]$$

where $\tilde{s}=r_{S}\left(e_{S}\left(s\right)\right)$, ${\tilde{x}}_{t}=r_{X}\left(e_{X}\left(x_{t}\right)\right)$. This loss is the difference between the actual data and the data encoded and recovered from the embedding space.

Unsupervised loss:

(14) $$L_{U}=E_{s,x_{1:T}∼p}[\log y_{S}+\sum_{t}\log y_{t}]+E_{s,x_{1:T}∼\hat{p}}[\log\left(1-{\hat{y}}_{S}\right)+\sum_{t}\log\left(1-{\hat{y}}_{t}\right)]$$

This is the loss in the traditional GAN model.

Supervised loss:

(15) $$L_{S}=E_{s,x_{1:T}∼p}[\sum_{t}{\left\|h_{t}-g_{X}\left(h_{S},h_{t-1},z_{t}\right)\right\|}_{2}]$$

where $h_{S}=e_{S}\left(s\right)$ and $h_{t}=e_{X}\left(h_{S},h_{t-1},x_{t}\right)$. This loss is the difference between the actual next-step latent vector and synthetic next-step latent vector.

Let $\theta_{e}$, $\theta_{r}$, $\theta_{g}$, and $\theta_{d}$, respectively, denote the parameters of the embedding, recovery, generator, and discriminator networks. The encoder and decoder are trained on both the reconstruction and supervised losses: $\underset{\theta_{e},\theta_{r}}{min}\left(\lambda L_{S}+L_{R}\right)$. The generator and discriminator are trained on supervised and unsupervised losses: $\underset{\theta_{g}}{min}\left(\eta L_{S}+\underset{\theta_{d}}{max}\right)L_{U}$.

#### 6.1.6. DoppelGANger [57]

DoppelGANger aims to generate time series data with high fidelity by capturing the complex correlations between measurements and metadata, maintaining long-term correlations and preventing mode collapse. To capture the correlations between metadata and measurements, DoppelGANger first generates metadata using an MLP generator, then it generates measurements using an RNN that takes the metadata as input. It also adopts an auxiliary discriminator which discriminates only on metadata to prevent the discriminator from handling a sample of high dimension. The losses from the two discriminators are combined by a weighting parameter α: $\underset{G}{min}\underset{D_{1},D_{2}}{max}L_{1}\left(G,D_{1}\right)+\alpha L_{2}\left(G,D_{2}\right)$, where $L_{i},i\in \left\{1,2\right\}$ is the Wasserstein loss of the original and the auxiliary discriminator, respectively. To maintain long-term correlations, it adopts LSTM and generates a batch of data with consecutive timestamps at each pass. To address the mode collapse problem for the measurements, it normalizes each time series signal individually, and stores the min/max as “fake“ metadata.

This work also identifies the fundamental challenges with both classical notions of privacy and recent advances to improve the privacy properties of GANs, and suggests a potential roadmap for addressing these challenges.

#### 6.1.7. COT-GAN [58]

Causal optimal transport generative adversarial network (COT-GAN) builds on optimal transport (OT) [80] theory, and constrains the transport plans to respect causality: the probability mass moved to the target sequence at time *t* can only depend on the source sequence up to time *t*. It uses Sinkhorn divergence with the causal constraint in OT theory to calculate the distance between synthetic and real time series data and makes it an item of the adversarial objective function. This work proposes a mixed Sinkhorn divergence which processes two batches at once to solve the convergence problem in the previous algorithm at the level of mini-batches. The discriminator consists of two separate neural networks parameterized using φ: $h_{\varphi_{1}}:={\left(h_{\varphi_{1}}^{j}\right)}_{j=1}^{J},\;M_{\varphi_{2}}:={\left(M_{\varphi_{2}}^{j}\right)}_{j=1}^{J}$. The adversarial objective function of COT-GAN is:

(16) $${\hat{W}}_{c_{\varphi }^{K},\epsilon }^{mix,L}\left(\hat{x},{\hat{x}}^{{}^{\prime }},{\hat{y}}_{\theta },{\hat{y}}_{\theta }^{{}^{\prime }}\right)-\lambda pM_{\varphi_{2}}\left(\hat{x}\right)$$

where $\hat{x}$ and ${\hat{x}}^{{}^{\prime }}$ are empirical measures corresponding to two samples of the dataset, and ${\hat{y}}_{\theta }$ and ${\hat{y}}_{\theta }^{{}^{\prime }}$ are the ones corresponding to two samples from the generator. λ is a positive constant and $pM_{\varphi_{2}}\left(\hat{x}\right)$ is the martingale penalization for $M_{\varphi_{2}}$.

Item $c_{\varphi }^{K}$ is a cost function whose output at time *t* depends on the input only up to time *t*:

(17) $$c_{\varphi }^{K}\left(x,y\right):=c\left(x,y\right)+\sum_{j=1}^{J}\sum_{t=1}^{T-1}h_{\varphi_{1},t}^{j}\left(y\right)\Delta_{t+1}M_{\varphi_{2}}^{j}\left(x\right)$$

Item ${\hat{W}}_{c_{\varphi }^{K},\epsilon }^{mix,L}\left(\hat{x},{\hat{x}}^{{}^{\prime }},{\hat{y}}_{\theta },{\hat{y}}_{\theta }^{{}^{\prime }}\right)$ is the mixed Sinkhorn divergence:

(18) $${\hat{W}}_{c_{\varphi }^{K},\epsilon }^{mix,L}\left(\hat{x},{\hat{x}}^{{}^{\prime }},{\hat{y}}_{\theta },{\hat{y}}_{\theta }^{{}^{\prime }}\right)=W_{c,\epsilon }\left(\hat{x},{\hat{y}}_{\theta }\right)+W_{c,\epsilon }\left({\hat{x}}^{{}^{\prime }},{\hat{y}}_{\theta }^{{}^{\prime }}\right)-W_{c,\epsilon }\left(\hat{x},{\hat{x}}^{{}^{\prime }}\right)-W_{c,\epsilon }\left({\hat{y}}_{\theta },{\hat{y}}_{\theta }^{{}^{\prime }}\right)$$

where $W_{c,\epsilon }\left(x,y\right)$ is the Wasserstein distance.

#### 6.1.8. TS-Benchmark [30]

TS-Benchmark is a benchmark of time series database. It contains a deep convolutional generative adversarial network (DCGAN)-based data generation model to generate large volumes of time series data from some real time series data. It first creates seed fragments from real time series data, and then generates synthetic fragments from real seeds using DCGAN. The generated fragments are connected to each other to generate a longer time series. The connectivity of a sequence *a* to another sequence *b* is defined as

(19) $$s\left(a,b\right)=\frac{1}{\sqrt{\sum_{i=1}^{l}{\left(a_{ti}-b_{hi}\right)}^{2}}}$$

where $a_{t}$ denotes the tail of sequence *a*, whose length is *l*, and $b_{h}$ denotes the head of sequence *b*, whose length is *l* too. A directed graph can be built by creating edges from a segment to others whose connectivity with the segment is greater than a threshold. The weight of a directed edge from segment *a* to segment *b* is

(20) $$w\left(a,b\right)=\frac{s\left(a,b\right)-\bar{s}}{\sum_{b^{{}^{\prime }}\in N_{a}}s\left(a,b^{{}^{\prime }}\right)-\bar{s}}$$

where $N_{a}=\left\{b|s\left(a,b\right)>\bar{s}\right\}$ and $\bar{s}$ is the previously mentioned threshold. Given an initial seed sequence *a*, a subsequent sequence *b* is generated by a random walk with probability w(a,b) on the directed graph. To connect these two sequences smoothly, $a_{t}$ and $b_{h}$ of the two adjacent sequences are spliced using a fitting function $c_{i}=\left(1-\sigma_{i}\right)a_{i}+\sigma_{i}b_{i}$, where $i=[-\frac{l}{2},\ldots ,\frac{l}{2})$ is the index of overlaps between sequences and σ is the sigmoid function $\sigma_{i}=\frac{1}{1+2^{-i}}$.

#### 6.1.9. RTSGAN [59]

The real-world time series generative adversarial network (RTSGAN) consists of an encoder–decoder module and a GAN. It first learns an encoder–decoder module which provides a mapping between a time series data instance and a fixed-dimension latent vector. Subsequently, it learns a generation module to generate vectors in the same latent space. The encoder, which takes dynamic and global features transformed into [0,1] as its input, is composed of an N-layer gated recurrent unit (GRU), a pooling layer, and a fully connected layer using LeakyReLU as the activation function. The decoder first reconstructs the global features via a fully connected layer, and then reconstructs the dynamic features via a GRU. The overall loss function of the encoder–decoder module is a linear combination of reconstruction loss for global features and dynamics features. The generation module uses an improved version of WGAN in which the generator aims to minimize the 1-Wasserstein distance between the real data distribution and synthetic data distribution with the help of an iteratively trained 1-Lipschitz discriminator.

Furthermore, this work proposes RTSGAN-M to address the value missing problem in time series data. RTSGAN-M adopts an observation embedding to enrich the information at each time step, and a decide-and-generate decoder which first determines the time and missing patterns of the next step and then generates the corresponding feature values based on both local and global dependencies.

### 6.2. VAE-Based Methods

#### 6.2.1. Variational Autoencoder

Autoencoder is an unsupervised algorithm used for feature extraction or data dimensionality reduction. It consists of an encoder and a decoder. The input features *x* are abstracted into intermediate variables *y* by the encoder, and then mapped back to the original data space $\bar{x}$ by the decoder, aiming to reconstruct the original data as accurately as possible. The purpose of an autoencoder is to extract abstract features *y*, and its learning process minimizes the loss function $L(x,\bar{x})$. The mean squared error function can be used: $L\left(x,\bar{x}\right)=\sum_{i=1}^{n}\left|\right|x_{i}-{\bar{x}}_{i}{\left|\right|}^{2}=\sum_{i=1}^{n}\left|\right|x_{i}-d\left(e\left(x_{i}\right)\right){\left|\right|}^{2}$. Where *i* represents the ith sample, and $x_{i}\in R^{n}$. Autoencoder can go from raw data *x* to abstract features *y*, which can achieve tasks such as data dimensionality reduction, denoising, compression, and feature extraction.

The autoencoder can reconstruct the intermediate variable *y* to $\bar{x}$. The variational autoencoder attempts to infer and learn the distribution of intermediate variable *y*, and generates data by sampling from *y*. It is a generative model. The variational autoencoder assumes that the posterior probability $q_{\phi }\left(y|x\right)$ follows a multi-dimensional mixture normal distribution, using two networks to estimate the mean and variance of hidden state $z^{\left(i\right)}$ corresponding to each sample. By regularizing the loss function with a regularization term, it ensures that $q_{\phi }\left(y|x\right)$ conforms to a standard normal distribution, ensuring the generative ability of the model.

#### 6.2.2. FSTS [60]

FSTS (few-shot learning for time series data generation) is a method for generating time series data based on autoencoders. Firstly, a small amount of data is used to pre-train the autoencoder. The encoder maps input data into the hidden space, $x_{h}=E\left(x_{in}\right)$, and then the decoder restores it back to the original space, $x_{out}=D\left(x_{h}\right)$, by minimizing mean square error between input and output data during training. Through pre-training, the autoencoder models the hidden space well enough for generating sufficient amounts of hidden space data from which large volumes of generated samples can be obtained using decoder restoration mechanisms. Although this method applies an autoencoder technique in data generation with good results achieved, it takes no specific design targeted at time series data and also fails to address problems related to regularity within the latent space.

#### 6.2.3. VRNN [61]

Variational RNN (VRNN) combines the methods of VAE and RNN by using latent-space random variables to represent time sequences, which explores for the first time the combination of VAE and RNN for generating sequence data. The process of generating data begins with calculating the prior probability based on the previous hidden state as historical information, then generating data by sampling from the prior probability, updating the hidden state, and calculating the posterior probability. The objective function of VRNN is basically consistent with that of the original VAE.

(21) $$E_{q(z_{\leq T}|x_{\leq T})}[\sum_{t=1}^{T}\left(-\mathrm{KL}(q\left(z_{t}|x_{\leq t},z_{<t}\right)∥p\left(z_{t}|x_{<t},z_{<t}\right))+\log p\left(x_{t}|z_{\leq t},x_{<t}\right)\right)].$$

#### 6.2.4. SRNN [62]

SRNN builds upon the basis of VRNN and combines RNN and SSM, which are commonly used methods in time series modeling. RNN is known for its strong non-linear fitting ability, but its hidden state is deterministic. On the other hand, SSM’s random state transition is more suitable for uncertainty modeling, but the inference process is usually simple. By integrating RNN and SSM, SRNN ensures that the random state does not affect the deterministic state during the generation process. During the inference process, SRNN incorporates a reverse RNN to capture future information.

(22) $$F_{i}\left(\theta ,\phi \right)=E_{q_{\phi }}[\log p_{\theta }\left(x_{1:T}|z_{1:T},{\tilde{d}}_{1:T}\right)]-KL\left(q_{\phi }\left(z_{1:T}|{\tilde{d}}_{1:T},x_{1:T},z_{0}\right)\;∥\;p_{\theta }\left(z_{1:T}|{\tilde{d}}_{1:T},z_{0}\right)\right)$$

#### 6.2.5. DSAE [63]

Compared to traditional autoencoders, the DSAE model introduces hidden variables that are invariant and variant over time, which can better handle temporal information in sequence data. Specifically, the DSAE model divides the hidden space into two parts: variant and invariant over time. The variable that varies with time represents the trend of data on the time axis, while the invariant variable represents features that remain unchanged on the time axis. This hierarchical architecture can distinguish between latent time-related features and those independent of time, helping to capture both static and dynamic characteristics of sequence data and further improving the model’s generative ability.

## 7. Challenges and Future Directions

Time series data generation methods still face the following challenges:

1. High dimensionality of time series: Time series data usually have high dimensionality, which increases the difficulty of training the time series data generation model. Generative models need to have sufficient memory capacity while avoiding overfitting to handle noise and discontinuities in the generation processes.
2. Long-term dependencies: Time series data usually have long-term dependencies, that is, previous data points may have an impact on the generation of subsequent data points. Many traditional generative models do not handle such long-term dependencies well, and for some time series data, such dependencies can be very important.
3. Insufficient data samples: In some fields, such as healthcare and finance, the data may be very limited and rarely labeled. Therefore, how to efficiently train generative models to generate high-quality time series data remains a challenging problem.

Different categories of methods face different challenges according to their properties. Learning-based methods mostly face all of the challenges, while other methods face challenges 1 and 2.

Future development directions of time series data generation methods may include the following aspects:

1. More efficient model design: At present, many time series data generation models have been proposed, but most of them require long training times and large amounts of computing resources. Future directions may include designing more efficient models to reduce the training time and computational cost.
2. Better long-term dependency modeling: Time series data usually exhibit long-term dependencies, where previous data points may have an impact on the generation of subsequent data points. Future directions may include designing better generative models that can efficiently handle long-term dependencies and maintain data continuity and smoothness during generation.
3. Time series data generation applications based on deep learning and advanced technologies: With the development of deep learning and other advanced technologies, future development directions may include the development of more time series data generation applications, such as speech synthesis, music generation, video generation, machine translation, natural language generation, etc. These applications can be combined with other technologies, such as automation, augmented reality, and virtual reality, etc., to create more intelligent, natural, and real time series data generation applications.

## 8. Conclusions

This paper introduces four categories of time series data generation methods in IoT, including rule-based methods, simulation-model-based methods, traditional machine-learning-based methods, and deep-learning-based methods. Among them, the rule-based methods are simple, fast, and highly controllable, but the generated results lack authenticity and cannot simulate complex system behavior so they can be used for scenarios in which the distribution of the generated data does not need to correspond to real data. The simulation-model-based methods can simulate the actual system behavior and are interpretable, but the data generation is limited by the specific situation in a particular field so they can be used for scenarios in which the distribution of the generated data is the same as a known stochastic process in the real world. The traditional machine-learning-based methods can handle more complex patterns of data and generate data faster, but the quality of data depends on the accuracy of the model and the quality of historical data. The deep-learning-based methods can learn higher-level features and generate more diverse and real data, but they require more computing resources, a larger volume of data, and longer processing time. These two categories of methods can be used for scenarios in which the distribution of the generated data is unknown beforehand and can be learned from real data.

Nowadays, time series data generation methods still face challenges in generating high-dimensional and long time series data. In addition, time series data generation methods need to solve problems such as insufficient data samples. Therefore, future research in time series data generation should focus on improving model efficiency, enhancing long-term dependency modeling, and following up the development of new deep learning technologies. We believe that the systematic classification and evaluation presented in this survey will be a valuable reference for researchers in the time series data generation field.

There are still several limitations of this survey. First, the classification is rough and lacks detailed classification, such as application-based classification. The scope of this survey does not include methods for time series data generation in a broad sense, such as text generation. The potential usages of big models such as GPT for time series data generation are also not discussed. These limitations will be the guidance for future studies.

## Figures and Tables

**Table 1:** Comparison of time series data generation methods.

Method	References	The Need for Real Data	The Need for Domain-Specific Knowledge	Controllability of Results	Authenticity of Results	Simulating Complex IoT System Behavior
Rule-based methods	[51,52,53]	×	×	Strong	Weak	Weak
Simulation-model-based methods	[37,45]	✓	✓	Strong	Depends on the model	Depends on the model
Traditional machine-learning-based methods	[29,32,39]	✓	×	Weak	Depends on the model	Strong
Deep-learning-based methods	[7,30,44,54,55,56,57,58,59,60,61,62,63]	✓	×	Weak	Strong	Strong

**Table 2:** Comparison of GAN-based methods.

Method	Reference	Support Condition/Metadata	Has Embedding	Other Features
C-RNN-GAN	[44]	×	×	
RCGAN	[7]	✓	×	”Train on synthetic data, test on real data“ strategy
SeqGAN	[54]	×	×	Modeling the generator as a RL policy
T-CGAN	[55]	✓(timestamp only)	×	Support for input data with missing value
TimeGAN	[56]	✓	✓	Combination of supervised and unsupervised loss
DoppelGANger	[57]	✓	×	Separating the generation and discrimination of metadata from time series data
COT-GAN	[58]	×	×	Utilization of causal optimal transport theory
TS-Benchmark	[30]	✓	×	Generating pieces of time series data and splicing them together
RTSGAN	[59]	✓	✓	Fixed length vector in the latent space; support for input data with missing value

## Data Availability

No new data were created or analyzed in this study. Data sharing is not applicable to this article.
